# Bacterial community structure across environmental gradients in permafrost thaw ponds: methanotroph-rich ecosystems

**DOI:** 10.3389/fmicb.2015.00192

**Published:** 2015-03-18

**Authors:** Sophie Crevecoeur, Warwick F. Vincent, Jérôme Comte, Connie Lovejoy

**Affiliations:** ^1^Département de Biologie and Takuvik Joint International Laboratory, Université LavalQuébec, QC, Canada; ^2^Centre d’Études Nordiques, Université LavalQuébec, QC, Canada; ^3^Institut de Biologie Intégrative et des Systèmes, Université LavalQuébec, QC, Canada; ^4^Québec Océan, Université LavalQuébec, QC, Canada

**Keywords:** bacterial diversity, methanotrophs, permafrost, pyrosequencing, 16S rRNA, thaw ponds

## Abstract

Permafrost thawing leads to the formation of thermokarst ponds that potentially emit CO_2_ and CH_4_ to the atmosphere. In the Nunavik subarctic region (northern Québec, Canada), these numerous, shallow ponds become well-stratified during summer. This creates a physico-chemical gradient of temperature and oxygen, with an upper oxic layer and a bottom low oxygen or anoxic layer. Our objective was to determine the influence of stratification and related limnological and landscape properties on the community structure of potentially active bacteria in these waters. Samples for RNA analysis were taken from ponds in three contrasting valleys across a gradient of permafrost degradation. A total of 1296 operational taxonomic units were identified by high throughput amplicon sequencing, targeting bacterial 16S rRNA that was reverse transcribed to cDNA. β-proteobacteria were the dominant group in all ponds, with highest representation by the genera *Variovorax* and *Polynucleobacter*. Methanotrophs were also among the most abundant sequences at most sites. They accounted for up to 27% of the total sequences (median of 4.9% for all samples), indicating the importance of methane as a bacterial energy source in these waters. Both oxygenic (cyanobacteria) and anoxygenic (Chlorobi) phototrophs were also well-represented, the latter in the low oxygen bottom waters. Ordination analyses showed that the communities clustered according to valley and depth, with significant effects attributed to dissolved oxygen, pH, dissolved organic carbon, and total suspended solids. These results indicate that the bacterial assemblages of permafrost thaw ponds are filtered by environmental gradients, and are complex consortia of functionally diverse taxa that likely affect the composition as well as magnitude of greenhouse gas emissions from these abundant waters.

## Introduction

One of the impacts of ongoing climate change is the northward migration of the limit of permafrost soils in subarctic landscapes, and this is leading to changes in the distribution and abundance of lakes and ponds caused by permafrost thawing and erosion (Vincent et al., 2013). These so called thaw ponds (thermokarst lakes and ponds) represent the most widespread aquatic ecosystem type in the circumpolar Arctic and Subarctic (Pienitz et al., 2008; Koch et al., 2014). In some northern regions of the Arctic, thaw lakes are disappearing as a result of evaporation and drainage (Smith et al., 2005), whereas in some southern locations such as subarctic Québec, Canada, permafrost lakes are expanding in size and numbers through increased permafrost thawing and erosion (Payette et al., 2004). Thaw lakes show cycles of expansion, erosion, drainage, and reformation (van Huissteden et al., 2011) that will likely accelerate under warmer climate conditions (Vincent et al., 2013).

Thawing permafrost has global implications for carbon biogeochemical cycling, since carbon that has been sequestered for 1000s of years becomes available for microbial degradation (Tranvik et al., 2009), resulting in the production of greenhouse gasses, especially carbon dioxide and methane. Despite this potential, greenhouse gas emissions from northern lakes and thaw ponds are often ignored in regional and global carbon budgets. These open waters potentially represent a source of around 24 Tg of methane emission per year (Walter et al., 2007). Methane is primarily produced by a few archaeal clades under anoxic conditions (Sowers, 2009), although methanogenesis has also been observed in oxic water columns (Grossart et al., 2011; Bogard et al., 2014). This biologically generated methane is available to aerobic methanotrophic bacteria that occur in the oxic zone or at oxic/anoxic boundaries (Borrel et al., 2011), and this methane oxidation activity may regulate net greenhouse gas emissions (Trotsenko and Khmelenina, 2005; Bodelier et al., 2013); for example, methanotrophy consumed up to 80% of the methane produced in a boreal Finnish lake (Kankaala et al., 2006). Thaw ponds can be either sources of greenhouse gas emissions (Walter et al., 2007; Laurion et al., 2010; Negandhi et al., 2013), or sinks for carbon sedimentation and storage (Walter et al., 2014), but reasons for these differences are poorly understood. Conditions that favor methanotrophy will have the net effect of decreasing methane release to the atmosphere, however, little is known about such processes in the abundant lakes and ponds on degrading permafrost landscapes.

Bacterial communities vary among lakes as a result of differences in physico-chemical and biological properties such as pH (Lindström et al., 2005), productivity and dissolved organic carbon (DOC) availability (Lindström and Leskinen, 2002; Yannarell and Triplett, 2004). For this reason even neighboring lakes in the same region may differ in their bacterial community structure (Casamayor et al., 2000; Van der Gucht et al., 2001). In addition, catchment and underlying soil properties vary, resulting in differences in the quantity and composition of organic matter entering lakes, which in turn may affect their bacterial communities (Judd et al., 2006; Kritzberg et al., 2006). Bacterial community composition in lakes also changes with depth (Shade et al., 2008; Garcia et al., 2013), especially in meromictic lakes where temperature, salinity, and oxygen gradients select for distinct communities down the water column (Hollibaugh et al., 2001; Comeau et al., 2012). Notably, a clone library study by Rossi et al. (2013) found distinct bacterial communities in surface and bottom waters of thaw ponds in northern Québec.

Arctic and sub-arctic ponds occur across a range of conditions that could select for particular bacterial taxa. For example, in northern Québec varied combinations of dissolved organic matter (DOM) and suspended particles result in striking differences in the optical properties of neighboring thaw ponds (Watanabe et al., 2011) and characteristic bacterial communities could be associated with the particular optical properties of individual ponds. The extent and stage of thawing of the surrounding permafrost could also potentially affect bacterial community structure; some thaw ponds occur in highly degraded permafrost while others are surrounded by more than 50% of intact permafrost. These different stages of permafrost thaw influence the landscape characteristics (e.g., vegetation cover, open water extent) and the geomorphology of the ponds (Bouchard et al., 2014), creating a gradient in concentrations of allochthonous DOM. With thawing more pronounced at the southern limits of permafrost, bacterioplankton in these regions would have greater access to this external supply of organic matter. Since microbial communities control biogeochemical processes such as methane balance, understanding factors that influence bacterial community composition is central for predicting net greenhouse gas emissions from the thawing landscape. Clone library analysis of four northern Québec ponds reported the presence of methanotrophs (Rossi et al., 2013), but little is known about how communities might vary over a range of permafrost conditions.

In addition, bacterial α-diversity (number of taxa per lake) could be influenced by local conditions such as depth, light availability, and pond productivity. For example, diversity is expected to be lower where low oxygen and light conditions select for a few specialist taxa (Shade et al., 2008). Lower primary productivity has also been linked with a lower diversity (Cardinale et al., 2006; Ptacnik et al., 2008), and light and nutrient limitation would therefore influence diversity. On a landscape scale, the diversity of animals and plants decreases at higher latitudes (Rahbek, 1995; Falge et al., 2002). However Fierer and Jackson (2006), found that for soil microbes α-diversity is independent of latitude but more related to soil properties, for example pH. If this were the case for thaw ponds then inherent properties of the landscape could also influence the α-diversity.

The goal of the present study was to investigate the diversity and distribution of potentially active bacterial communities in subarctic thaw ponds across a gradient of limnological conditions and permafrost, from discontinuous permafrost in the North to sporadic permafrost in the South. We applied high throughput sequencing, targeting the V6–V8 hypervariable region of 16S rRNA. The use of this hypervariable region enabled us to identify community assemblages in three geographically separate valleys that differed in their underlying stage of permafrost degradation. Specifically we tested the hypotheses that: (1) different bacterial communities develop at the surface and bottom of each thaw pond because of the strong physico-chemical gradient through the water column; (2) bacterial community composition varies across the gradient of permafrost thawing and degradation; and (3) given the known high concentrations of methane in these waters (Laurion et al., 2010), methanotrophic bacteria are well-represented in thaw pond bacterial assemblages.

## Materials and Methods

### Study Sites and Sampling

**FIGURE 1:**
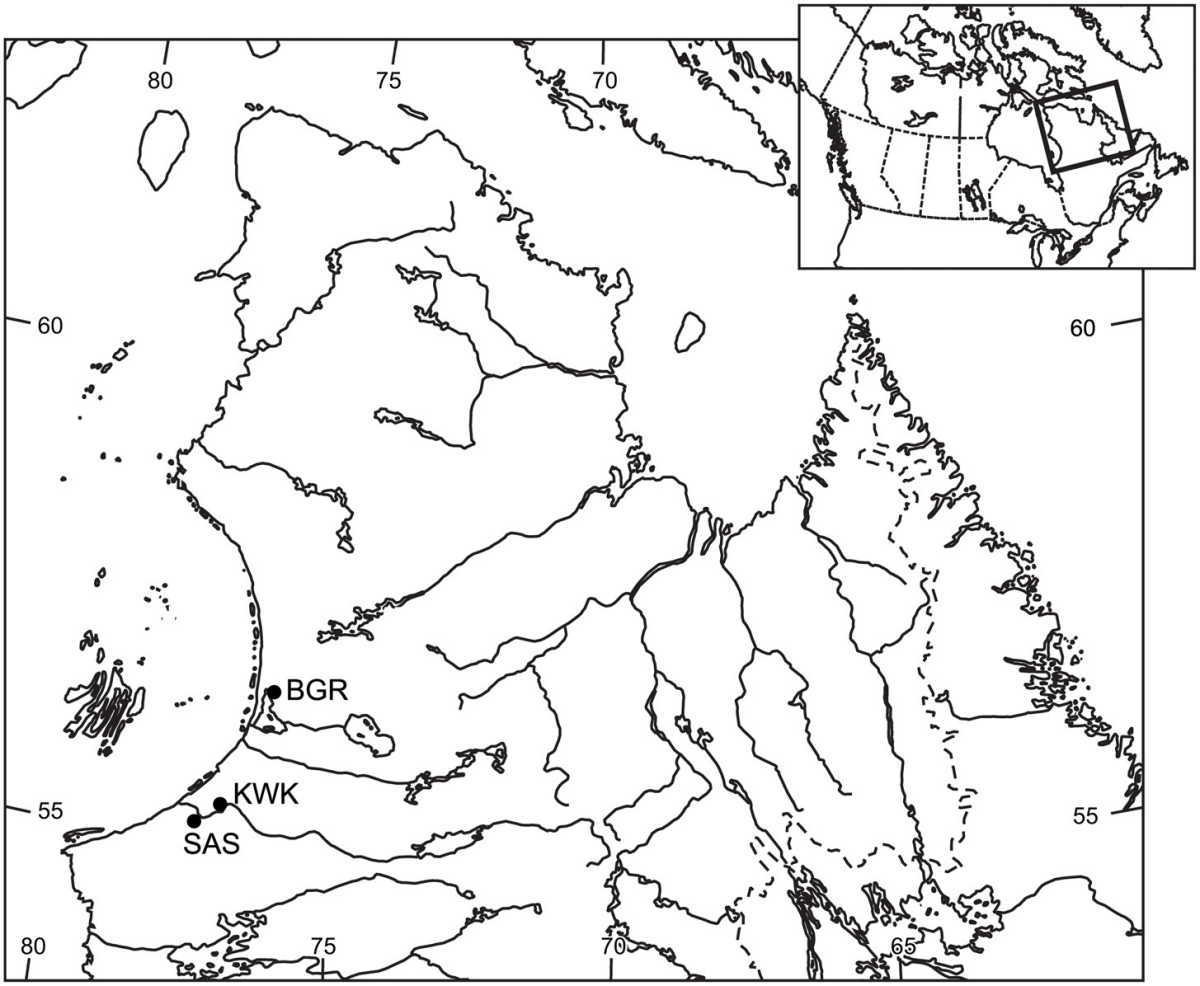
**Location of the three sampling valleys in Nunavik, subarctic Québec, Canada**.

Samples were collected from 1 to 13 of August 2012 from three different subarctic valleys (**Figure 1**), which were chosen to represent a gradient of thawing permafrost. Ponds from these valleys have been investigated since 2006 and nomenclature follows that of earlier studies (Calmels et al., 2008; Breton et al., 2009; Laurion et al., 2010; Watanabe et al., 2011). The ponds were designated with a three letter prefix and a unique number or number letter combination. The KWK (55∘16^′^N; 77∘46^′^W) and SAS (55∘13^′^N; 77∘42^′^W) ponds are in two separate river valleys close to the village of Whapmagoostui-Kuujjuarapik in the sporadic permafrost zone, while the BGR ponds (56∘37^′^N; 76∘13^′^W) are in the Sheldrake River valley close to the village of Umiujaq, Québec, in the discontinuous permafrost region. The ponds have formed in thawing permafrost mounds that are primarily organic (peat) or mineral; the term palsa refers to organic mounds and lithalsa to mineral mounds (Gurney, 2001; Calmels et al., 2008). KWK and BGR ponds originated from the thawing of lithalsas and SAS ponds from palsas. BGR and KWK ponds are surrounded by shrubs (*Salix planifolia* and *Betula glandulosa*) and sparse trees (*Picea mariana*, *Picea glauca*, *Larix laricina*; Calmels et al., 2008; Breton et al., 2009). The SAS valley vegetation, in contrast, is dominated by *Carex* and *Sphagnum* (Arlen-Pouliot and Bhiry, 2005; Bhiry et al., 2011). Two ponds were selected from the BGR valley (1 and 2), three from the KWK valley (1, 6, and 23) and two from the SAS valley (1B and 2A; **Table 1**). Ponds were sampled from an inflatable boat held near the center of the ponds using ropes tethered to the shore. Temperature, conductivity, dissolved oxygen (DO), and pH profiles were taken using a 600R multiparametric probe (Yellow Spring Instrument). Surface water samples were collected directly into submerged acid and sample rinsed 4-L Cubitainers and near-bottom samples were collected using a horizontally mounted Van Dorn bottle (Wilco) and immediately transferred to 4-L Cubitainers. Care was taken to overfill the Cubitainers when sampling the bottom water and all the Cubitainers were capped to minimize exchange with the atmosphere. The filled Cubitainers were then transported back to the laboratory by helicopter and processed within 2 h.

**Table 1:** **Limnological properties of the sampled thaw ponds: dissolved oxygen (DO), DO % saturation (% sat.), conductivity (Cond), chlorophyll* a* (Chl* a*), dissolved organic carbon (DOC), total suspended solids (TSS), soluble reactive phosphorus (SRP), and total nitrogen (TN)**.

Pond	Depth (m)	DO (mg L^--1^)	DO (% sat.)	pH	Cond (μS cm^--1^)	Chl *a* (μg L^--1^)	DOC (mg L^--1^)	TSS (mg L^--1^)	SRP (μg L^--1^)	TN (mg L^--1^)
SAS1B	0	6.37	63	6.0	93	4.9	15.5	27	2.7	0.9
	1	1.54	15	5.63	212	2.9	16.2	33	3	1.8
SAS2A	0	5.75	64	6.2	97	1.7	14.9	2.6	3.1	0.7
	2.4	0.26	2	5.58	300	18.1	18.9	16	4.1	1.6
KWK1	0	9.69	101	6.66	63	10.9	12	26	3.7	0.6
	1.8	0.50	4.2	6.22	150	10.3	12	141	12.6	1.0
KWK6	0	9.94	97	6.36	82	3.3	5.2	8.2	1.3	0.4
	3	1.82	17	6.35	112	27.1	5.2	16	1	0.7
KWK23	0	9.8	97	6.44	29	1.9	7.8	8.3	5.5	0.4
	3.2	0.36	2.7	6.09	259	7.2	10.9	74	133.6	2.7
BGR1	0	10.0	101	7.38	168	0.9	3.5	2.4	2.4	0.2
	3.2	4.06	37	7.56	190	1.1	3.3	3.8	2.2	0.4
BGR2	0	9.43	94	7.31	209	2.4	9.3	13	3.4	0.4
	1	3.47	34	7.17	387	3.8	8.7	57	4.5	1.2

*The surface samples correspond to 0 m and bottom samples to the second depth for each pond.*

### Physico-Chemical Analysis

Water samples for DOC, soluble reactive phosphorus (SRP), and major ion analysis were filtered through a MilliQ water pre-rinsed 47-mm diameter 0.22-μm pore size acetate filters (Whatman). DOC concentrations were analyzed using a Shimadzu TOC-5000A carbon analyzer calibrated with potassium biphthalate. Three blank filters of Milli-Q water passed through the filters were analyzed along with the samples and these small background values were subtracted. Water samples for total phosphorus (TP) and total nitrogen (TN) were preserved with H_2_SO_4_ (0.15% final concentration) and analyzed using standard methods (Stainton et al., 1977) at Institut National de la Recherche Scientifique (Quebec City, QC, Canada). Total suspended solids were collected by filtration onto preweighed 47 mm GF/F filters (Whatman) that had been precombusted at 500∘C for 4 h. The GF/F filters were then oven dried for 2 h at 60∘C and reweighed. Samples for chlorophyll *a* (Chl *a*) were filtered onto a GF/F 25 mm filters (Whatman) and stored at -80∘C. Chl*-a* concentrations were determined using high performance liquid chromatography (ProStar HPLC system, Varian, Palo Alto, CA, USA) following the protocol of Bonilla et al. (2005).

### RNA Collection and Extraction

Water samples were first prefiltered through a 20-μm mesh to remove larger organisms and then filtered sequentially through a 3-μm pore size, 47-mm diameter polycarbonate filter (DHI lab product) and a 0.2 μm Sterivex unit (Millipore) with a peristaltic pump. From 100 to 600 mL of water were filtered and the filtration was stopped after 2 h to minimize RNA degradation. The size fractionation was employed to distinguish between particle-attached bacteria on the 3-μm filter and free-living bacteria on the 0.2-μm filter (Crump et al., 1999; Galand et al., 2008; Mohit et al., 2014). Both filters were preserved in RNAlater (Life Technologies) and the filters were stored at -80∘C until processed. The PowerWater Sterivex DNA Isolation Kit was used for extracting the RNA from the Sterivex units for the BGR1, KWK6 surface, KWK23 surface, and SAS1B surface. The large fraction for the same samples was extracted using the PowerWater DNA Isolation kit (MoBio). Protocols were adapted for RNA analysis by adding 1–2% (final concentration) of β-mercaptoethanol as a preliminary step. The extraction column was loaded with 50% ethanol (final concentration) to fix the RNA to the column. RNase-free water was used for the final elution step. The co-extracted DNA was then digested with the RTS DNase Kit (MoBio). Following problems with potential polyphenol contamination in some samples, the remaining samples were extracted with the AllPrep DNA/RNA Mini Kit (Qiagen). This protocol was modified by the addition of cross-linked polyvinylpyrrolidone (PVP, Alfa Aesar) to a final concentration of 10% before loading the samples onto the lysate homogenization column. Prior to use, the PVP was sterilized with UV light and was then added as a reagent to remove potential contaminating organic matter (humic acids and polyphenols). For all samples, the extracted RNA was converted to cDNA immediately with the High Capacity cDNA Reverse Transcription Kit (Applied Biosystems-Ambion). The concentrations and quality of cDNA were checked on a 1% agarose gel; cDNA was not detected from the large fractions of KWK23 and the two SAS ponds, and these samples were therefore not further processed. All cDNA was stored at -80∘C until analysis.

### High Throughput Multiplex Tag Sequencing

The V6–V8 region of the bacterial 16S rRNA that had been converted to cDNA was amplified using the 454 primers as described in Comeau et al. (2011). PCR was carried out in a total volume of 20 μL; the mixture contained HF buffer 1X (NEB), 0.25 μM of each primer, 200 μM of each dNTPs (Life Technology), 0.4 mg mL^-1^ BSA (NEB), 1 U of Phusion High-Fidelity DNA polymerase (NEB), and 1 μL of template cDNA. Two more reactions with 5X and 10X diluted template were also carried out for each sample, to minimize potential primer bias. Thermal cycling began with an initial denaturation at 98∘C for 30 s, followed by 25 cycles of denaturation at 95∘C for 10 s, annealing at 50∘C for 30 s, extension at 72∘C for 30 s and a final extension at 72∘C for 420 s. The three dilution reactions were pooled and purified with a magnetic bead kit Agencourt AMPure XP (Beckman Coulter) and then quantified spectrophotometrically with the Nanodrop 1000 (Thermo Fisher Scientific). The amplicons were sequenced on two 1/8 plates of the Roche 454 GS-FLX using the “PLUS” chemistry at the IBIS/Laval University, Plate-forme d’Analyses Génomiques (Québec, QC). Raw 454 sequences have been deposited in the NCBI database under accession number SRP050189.

### Sequence Processing and Statistics

Sequences were analyzed using the UPARSE pipeline (Edgar, 2013). For quality filtering, the sequences were truncated at 340 and 300 bp for the first and second 1/8 plate runs to keep 75% of the reads at the 0.5 expected error rate. Singletons as well as chimeras were then removed and operational taxonomic units (OTUs) were determined at the ≥97% similarity level. Taxonomic assignment of these OTUs was performed using the Mothur classifier (Schloss et al., 2009) with a 0.8 confidence threshold based on the SILVA reference database (Pruesse et al., 2007) modified to include sequences from our in-house, curated northern 16S rRNA gene sequence database. Shannon, Simpson, and Chao1 diversity indexes were estimated for each sample using quantitative insights into microbial ecology (QIIME) pipeline (Caporaso et al., 2010) by creating multiple rarefaction statistics. Species richness and evenness were estimated with Shannon and Simpson indexes while the Chao1 index provides an estimate of true richness (Colwell, 2012). Three-way analysis of variance (three-way ANOVA) was used to assess differences in the diversity indices between valleys, fractions, and depths. As the Simpson index is a proportion, the data were arcsin transformed to achieve normality. An a posteriori Tukey HSD test was run to identify differences between valleys.

β-diversity analysis was used as a means of comparing ponds; for this analysis the dataset was re-sampled to ensure the same number of reads per sample (3071), which corresponded to the sample with the fewest number of sequences. β-diversity was derived using the command multiple_rarefaction_even_depth.py in QIIME. The rarefaction curve for OTUs reached a plateau between 2000 and 4000 reads, indicating that our selection of 3071 reads provided a reasonable representation of the community.

Downstream statistical analyses were performed in R (version 3.0.1; R Core Team, 2013) using the package Vegan (Oksanen et al., 2013). The community data matrix was square root transformed before running the statistical analyses. β-diversity was assessed by the use of Bray–Curtis dissimilarity to estimate compositional difference among sampling sites. Distance-based redundancy analysis (dbRDA) was performed to test and identify the influence of environmental variables on the composition matrix. For the latter analysis, explanatory variables were selected via a forward selection and highly correlated variables were removed from the analysis to ensure that colinearity would not reduce analysis quality. The variance inflation factors (VIFs) were calculated for each variable and none of the coefficients exceeded the value of 5, we note that multicollinearity should be examined when the VIF exceeds 10 (Borcard et al., 2011). Compositional differences among valleys, depths, and fraction were tested with permutation tests (999 permutations) on the Bray–Curtis metric using the function Adonis in the Vegan package (Oksanen et al., 2013).

We defined ‘bacterial dominants’ as the 10 most abundant OTUs from each valley. Each OTU was submitted to a separate BLASTn search in NCBI GenBank (http://blast.ncbi.nlm.nih.gov/Blast.cgi) to identify the nearest match. In some cases a single OTU corresponded to two separate genera. We constructed a reference tree using longer sequences from clone libraries and GenBank to resolve these uncertainties. The short reads were mapped onto the reference tree using the ParInsert command available in QIIME. In many cases, several different OTUs had matches to a single genus.

## Results

### Limnological Conditions

**FIGURE 2:**
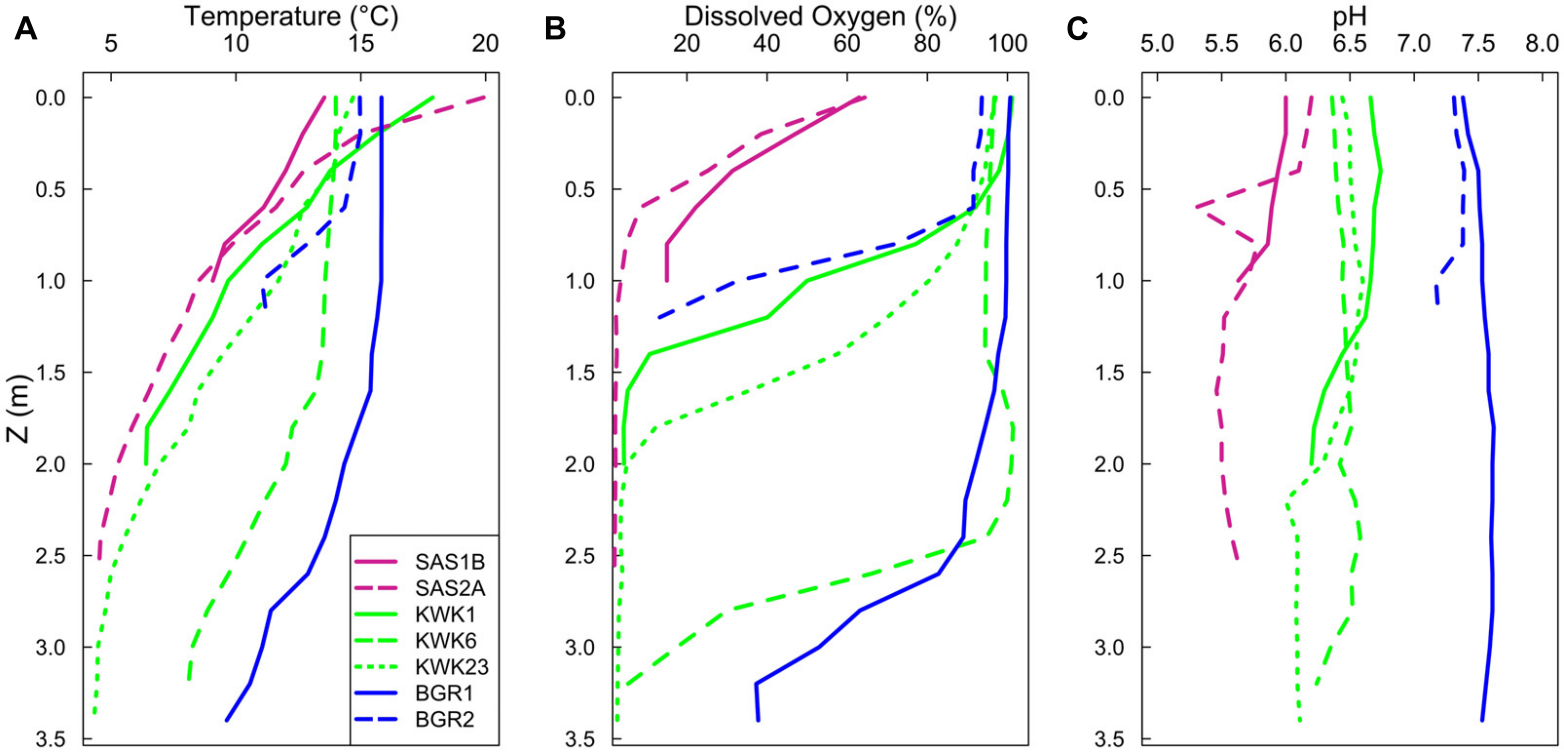
**Profiles of temperature **(A)**, dissolved oxygen **(B)**, and pH **(C)** as a function of the depth for the seven studied ponds**.

Temperatures decreased down the water column, with markedly colder bottom waters in all of ponds (**Figure 2A**). The surface waters were well-oxygenated except for the SAS sites, which had only 5–6 mg L^-1^ of DO at the surface (**Table 1** and **Figure 2B**). DO concentrations fell to lower values with depth (**Figure 2B**). Ponds were hypoxic (<2 mg L^-1^) at the near-bottom sampling depths, with the exception of the bottom of the BGR ponds where DO levels were around 3–4 mg L^-1^ (**Table 1**). There was a pH gradient across the three valleys: the SAS valley ponds were the most acidic with pH values from 5.3 to 6.2, while those in the KWK valley ranged from 6.0 to 6.7 and in the valley from 7.1 to 7.5. In all ponds, pH decreased slightly with the depth (**Figure 2C**). The other measured limnological variables also showed large differences between valleys, ponds, and depths (**Table 1**). Conductivity was higher in the bottom waters, by up to an order of magnitude in KWK23. DOC concentrations varied from <4 mg L^-1^ in the blue–green colored surface waters of BGR1 to >15 mg L^-1^ in the black SAS ponds. The total suspended solids varied by up to an order of magnitude, even among ponds from the same valley; (for example, SAS1B versus SAS2A and BGR1 versus BGR2), and concentrations increased with depth in all ponds. The indicators of trophic state (Chl *a*, SRP, and TN) were mostly in the oligotrophic to mesotrophic range, with higher values near the bottom of some the ponds, notably KWK23 (SRP and TN), and SAS2A and KWK6 (Chl *a*).

### Bacterial α-Diversity

The sequencing statistics for individual ponds are given in Table S1. For the overall study, 112,479 sequences (reads) were obtained, yielding a total of 1296 OTUs (excluding singletons, defined as OTUs that only occurred once in the entire data set). Three-way ANOVA for the different sample groupings (**Table 2**) showed that there was no significant difference in α-diversity as estimated by either the Shannon or Simpson indices between depths, size fractions, or valleys. However, the Chao1 index showed significant differences between valleys (*p* = 0.018) and fraction (*p* = 0.004), and a significant interaction between valleys and depth (*p* = 0.01). The mean species richness of the KWK ponds was 32% higher than in the BGR ponds, and the Tukey HSD test showed that the significant difference between valleys was only between KWK and BGR (*p* = 0.015). The mean Chao1 index for the large fraction was 22% greater than for the small fraction (**Table 2**).

**Table 2:** **Sequencing and diversity statistics for samples grouped according to valley, depth, or size fraction**.

Sample group	OTUs^a^	Shannon^a^	Simpson^a^	Chao1^a^
SAS	183 (11)	4.35 (13)	0.88 (4)	210 (8)
KWK	211 (27)	5.09 (10)	0.91 (8)	224 (23)
BGR	156 (39)	4.58 (16)	0.89 (6)	170 (38)
Surface	176 (26)	4.98 (14)	0.92 (5)	194 (25)
Bottom	196 (34)	4.56 (13)	0.87 (7)	210 (31)
Small	169 (34)	4.66 (14)	0.90 (7)	187 (30)
Large	216 (21)	4.96 (13)	0.92 (7)	229 (22)

*Values are means (*n*= 3 to 14) with CV (SD as % mean) in parentheses. ^a^Calculated with an OTU definition of 97% similarity.*

### Bacterial β-Diversity and Community Composition

**FIGURE 3:**
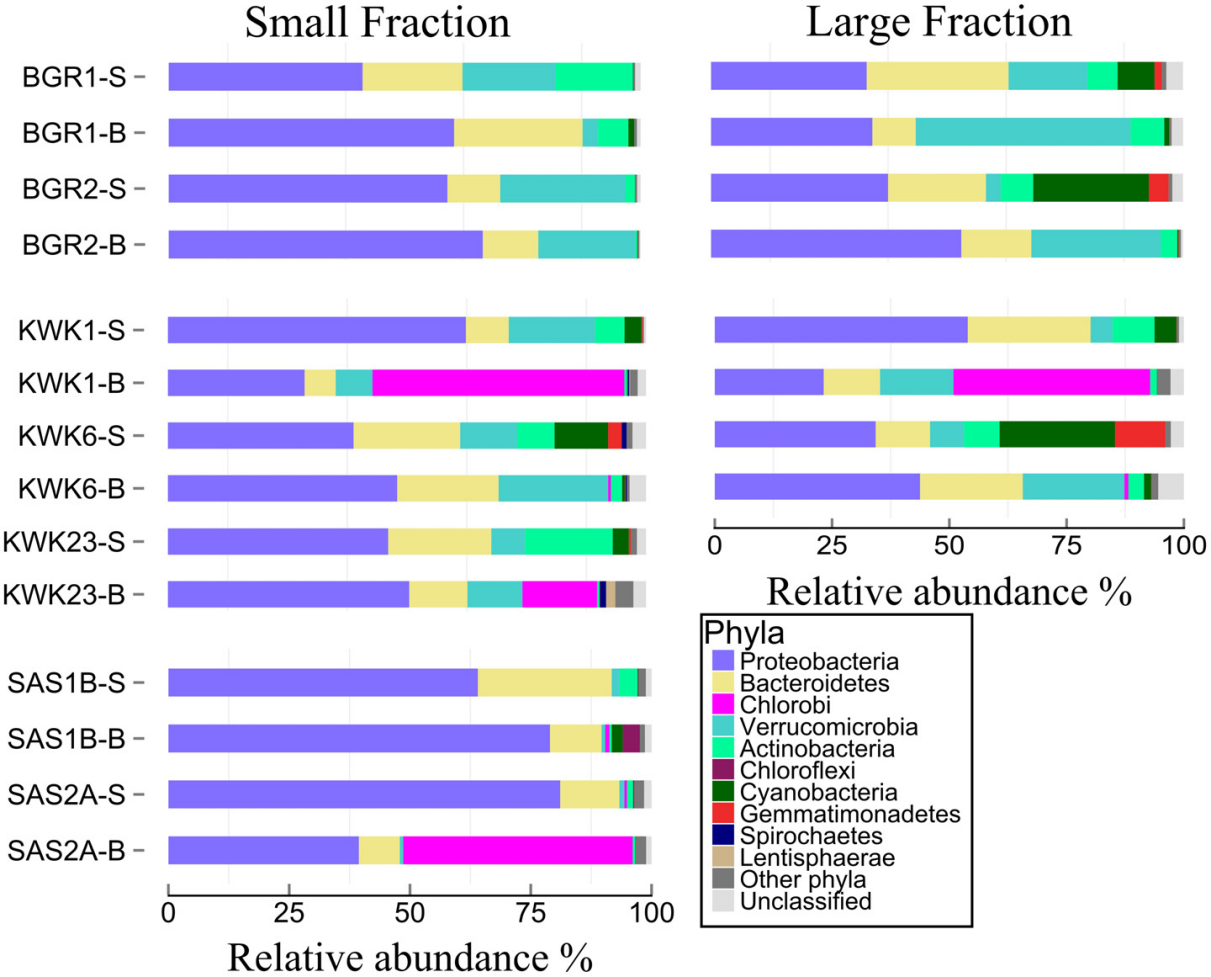
**Relative abundance of the different phyla.** The samples were from the surface (-S) and bottom (-B) of ponds in the three valleys. The small fraction (left) is for samples in the size range 0.2–3 μm, and the large fraction is for 3–20 μm. Phyla that were less than 1% of total abundance are combined under “Other phyla.”

All ponds were dominated by Proteobacteria followed by Bacteroidetes, Verrucomicrobia, and Actinobacteria (**Figure 3**). The maximum Proteobacteria representation (80% of reads) was in the SAS2A surface community. Within the Proteobacteria, β-proteobacteria was the dominant class, except at the bottom of KWK23 where γ-proteobacteria dominated. Chlorobi dominated the bottom of KWK1 and SAS2A. *Gemmatimonas*, Lentisphaerae and *Spirochaetes* were found in low abundance (about 1% of the community) within the KWK valley. There was little difference between the small and large fractions, with the exception of the BGR1 bottom community that had a high proportion (25% of reads) of cyanobacteria in the large fraction only.

### Bacterial Dominants and High Abundance of Methanotrophs

A total of 21 separate taxa were found in the top 10 OTUs from each valley (**Table 3**), which accounted for 38% of the total number of reads. The two most abundant OTUs corresponded to two β-proteobacteria, *Variovorax* and *Polynucleobacter*. These two taxa were in all samples, and accounted for up to 30% of the community reads in each sample. Another OTU in the family Puniceicoccaceae (Verrucomicrobia) was more abundant at the BGR valley and represented up to 40% of the reads in the surface large fraction of BGR2. One OTU of *Pelodictyon* (Chlorobiaceae) represented up to 50% of the bottom community reads for KWK1 and another OTU of *Pelodictyon* was the most abundant taxon in the bottom of SAS2A.

**Table 3:** **Identity of the 10 most abundant OTUs (defined at a level of 97% similarity) in each valley following the SILVA taxonomy**.

Number of reads	Silva taxonomy	% Identity	Isolation source	Accession number	GenBank taxonomy
7461	Comamonadaceae	100	Wheat phyllosphere	KF054966	*Variovorax soli*
6253	*Polynucleobacter*	100	Lake Grosse Fuchskuhle	KC702668	*Polynucleobacter*
5757	Puniceicoccaceae	100	Yellowstone Lake	HM856500	Opitutae
3748	*Pelodictyon*	99	Lake chemocline	AM086645	*Pelodictyon clathratiforme*
2454	*Methylobacter*	99	Thaw pond hypolimnion	JN656724	uncultured gamma proteobacterium
1881	*Rhodoferax*	100	Soil	GQ421098	uncultured *Rhodoferax*
1539	*Rubrivivax*	99	Foodplain	FM886868	*Rubrivivax gelatinosus*
1317	Comamonadaceae	99	Waterfall	KM035968	*Curvibacter*
1275	ACK-M1	100	Irrigation water	JX657295	uncultured actinobacterium
1260	Nostocaceae	99	Eutrophic pond	FN691914	*Dolichospermum curvum*
1256	*Sediminibacterium*	99	Yellowstone Lake	HM856392	uncultured *Sediminibacterium*
1250	*Sediminibacterium*	100	Lake epilimnion	HQ532649	uncultured Bacteroidetes
1137	*Pelodictyon*	97	Lake chemocline	AM086645	*Pelodictyon clathratiforme*
1085	*Limnohabitans*	99	Daphnia Digestive tract	HM561466	uncultured *Limnohabitans*
1003	*Sediminibacterium*	99	Yellowstone Lake	HM856387	uncultured *Sediminibacterium*
947	*Methylotenera*	99	Biodeteriorated wood	KC172609	uncultured Methylophilaceae
761	Chitinophagaceae	99	Lake epilimnion	HQ532140	uncultured Bacteroidetes
739	Synechococcales	99	Meromictic lake	AB610891	*Synechococcus*
637	*Methylobacter*	100	Thaw pond hypolimnion	JN656784	uncultured gamma proteobacterium
477	Methylococcaceae	96	Landfill cover soil	HF565143	*Methylobacter*
450	*Polaromonas*	99	Stems	KF385223	uncultured *Polaromonas*

*Following a BLASTn search, nearest matches and the providence of representative reads in GenBank were identified. Several groups appear multiple times because different OTUs match the same group. See **Figure 5B** for their distribution.*

**FIGURE 4:**
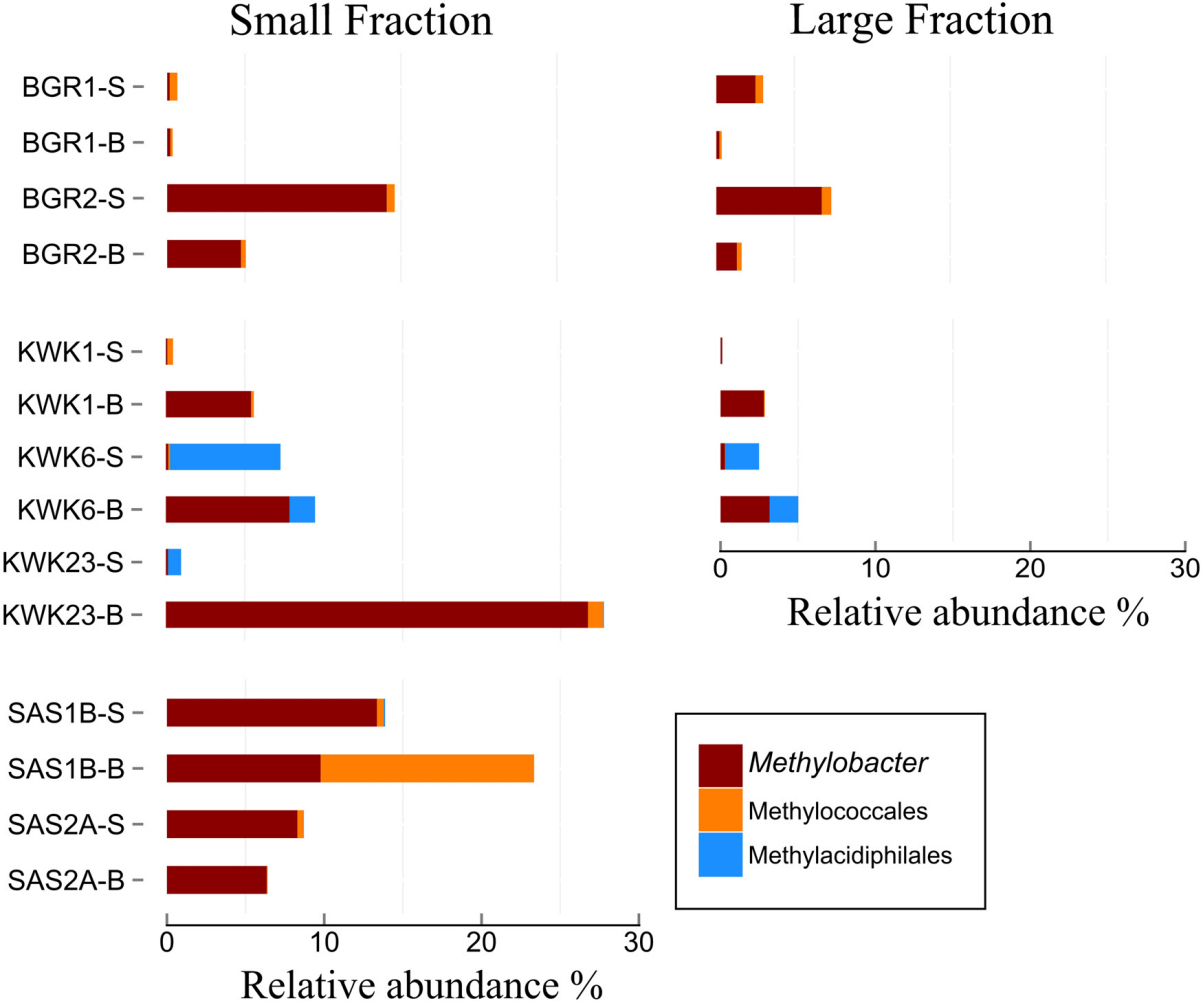
**Relative abundance of methanotrophic bacteria.** The samples were from the surface (-S) and bottom (-B) of ponds in the three valleys. The small fraction (left) is for samples in the size range 0.2–3 μm, and the large fraction is for 3–20 μm. The taxa are labeled according to the highest taxonomical level; genera that represented less than 1% of total abundance were grouped together and labeled by their shared order.

**FIGURE 5:**
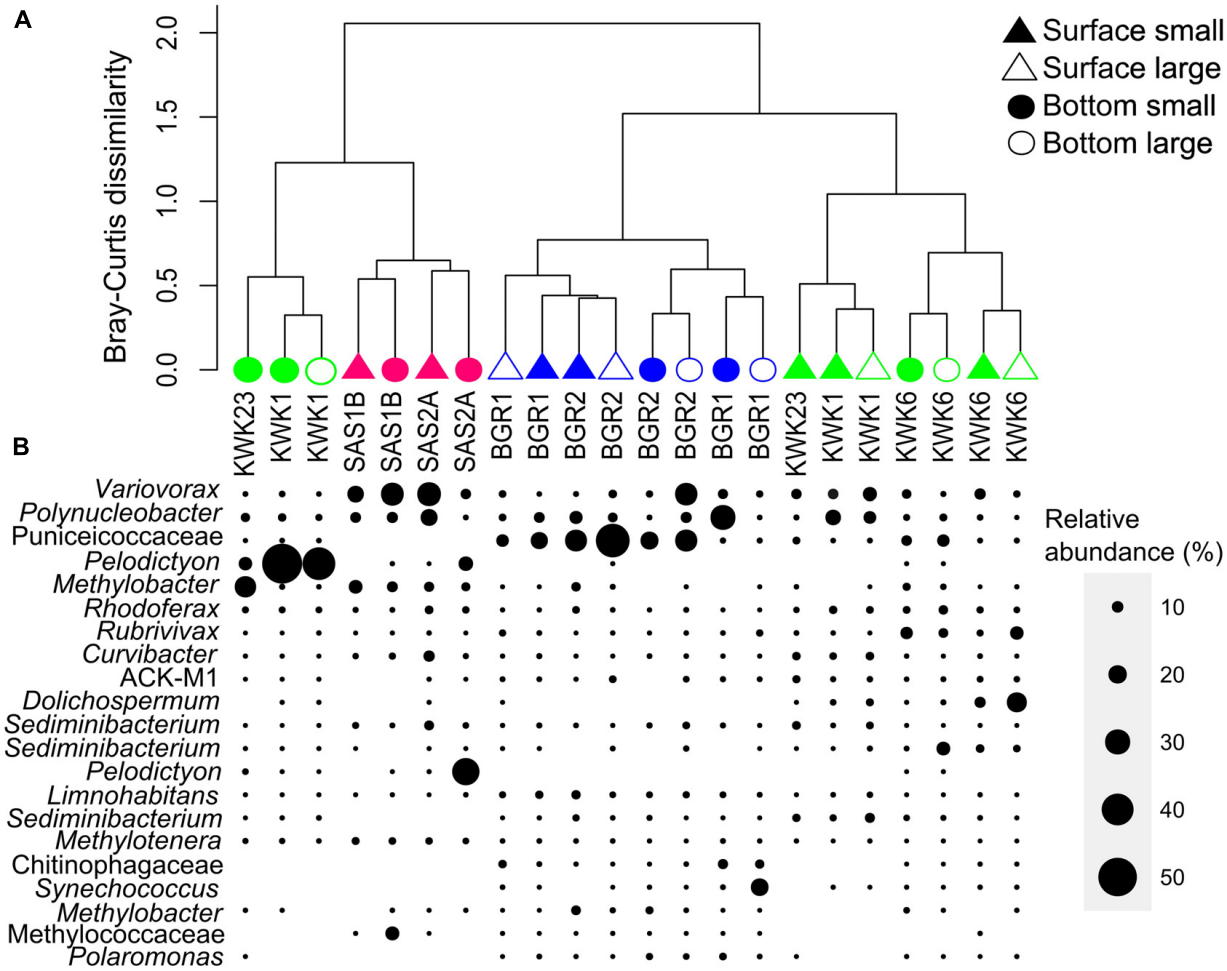
**(A)** Bray–Curtis dissimilarity cluster analysis with the community data matrix (OTUs clustering at >97% of identity) for the study ponds. Surface samples are represented by triangles and bottom samples by circles, either filled (small fraction) or open (large fraction). **(B)** The bacterial dominants in each sample identified by their lowest taxonomical level either found on GenBank or SILVA (see **Table 3**); the size of the filled circle is proportional to their relative abundance.

Finer taxonomic analysis revealed the presence of methanotrophic bacterial groups at all sites (**Figure 4**), with 0.1–27% of OTUs matching *Methylobacter* (5% on average per site). Other methanotrophic bacteria belonging to the family Methylococcales (*Methylocaldum*, *Methylomonas, Crenothrix*, or unknown Methylococcales) were found in all ponds, although sometimes in low proportions (<1%). An exception was in the bottom waters of SAS1B where methanotrophs accounted for 23% of the sequences, more than half of which were in the Methylococcales. Sequences corresponding to a newly discovered order of methanotrophic bacteria, Methylacidiphilales (Verrucomicrobia), were found at the KWK and SAS valleys, in smaller proportions compared to other methanotrophs; for example, 1–6% of the total reads in the KWK6 surface samples. Amongst the dominant OTUs, methanotroph sequences were relatively abundant in most samples (**Figures 4 and 5**), with a median representation of 4.9% of total reads per sample, and a maximum of 25% for one single *Methylobacter* OTUs in the KWK23 bottom community reads. Another OTU corresponding also to *Methylobacter* was present at the BGR valley along with the OTU belonging to the order Methylococcaceae.

Some of the most abundant OTUs matching genera *Rhodoferax*, *Rubrivivax*, *Limnohabitans, Curvibacter*, the Actinobacteria ACK-M1, the three *Sediminibacterium* OTUs and the methylotrophic genus *Methylotenera* were distributed evenly across samples. The filamentous cyanobacterium *Dolichospermum* was highly abundant only in the surface of KWK1 and KWK6 where it contributed 20% of the community. Finally, OTUs matching *Synechococcus, Polaromonas* and a taxon in the Chitinophagaceae (Bacteroidetes) were more abundant in samples from the BGR and KWK valleys (**Figure 5B**).

### Bacterial Community Structure as a Function of Environmental Gradients

Community structure mostly followed a regional pattern as ponds from the same valley clustered together in the Bray–Curtis dissimilarity dendrogram (**Figure 5**). There were some exceptions, with the bottom of KWK 1 and 23 clustering closer to the SAS sites and apart from the other KWK samples. Within the BGR cluster, surface and bottom communities clustered separately while the two depths clustered together at the SAS sites. In terms of community composition, with the exception of the more aerobic BGR waters, the bottom communities were strikingly different from those at the surface because of the presence of anoxygenic phototrophs, specifically Chloroflexi and Chlorobi. Small and large fractions from the same sample always clustered together. A permutation test was used to test for differences between valleys, depth and size fraction and indicated significant differences in community composition according to valley (*p* = 0.001) and depth (*p* = 0.009) as well as a significant interaction between valleys and depth (*p* = 0.011), indicating that the difference between surface and bottom communities depended on valley location. Pairwise comparison indicated that amongst the significant differences between sites, communities in the KWK valley were significantly different from those in the BGR and SAS valleys (*p* = 0.001 and *p* = 0.015) but BGR and SAS were not significantly different. Permutation tests were carried out to compare surface versus bottom communities, and all four dominant phyla (Proteobacteria, Bacteroidetes, Verrucomicrobia, and Actinobacteria) showed significant differences between depths (*p* < 0.05). There were no significant differences between fractions (*p* = 0.5).

**FIGURE 6:**
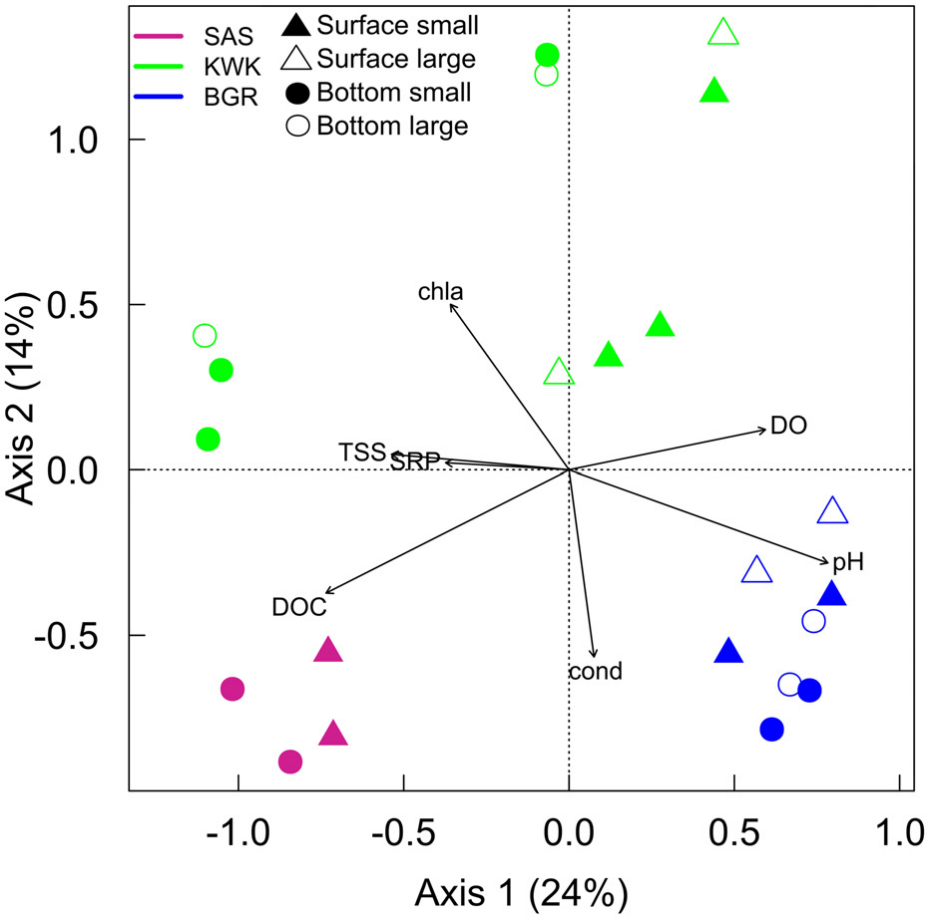
**Distance-based redundancy analysis ordination plot showing selected environmental variables that were significantly correlated with sample distribution.** Abbreviations for the environmental variables are given in **Table 1**

The dbRDA ordination (**Figure 6**) explained 64% of the variation and confirmed the regional pattern. The first horizontal axis was significantly correlated with pH, DO, TSS, and DOC while the vertical axis was significantly correlated with Chl* a* and conductivity. KWK samples were more dispersed compared to the SAS and BGR samples. The distribution of the BGR sites was mostly explained by the higher pH in this valley. The BGR sites and all of the surface KWK samples were separated from the other samples including bottom layers from KWK ponds and appeared associated with the higher concentrations of DO, while the bottom of KWK1 and 23, the SAS sites and the bottom of KWK6 were at the low end of the oxygen gradient. The SAS valley was differentiated by its higher DOC, and the bottom samples from KWK1 and KWK23 by their higher concentrations of TSS and SRP. The surface of the KWK sites and KWK6 bottom were characterized by lower conductivity and higher Chl *a*.

## Discussion

### Bacterial α-Diversity

The subarctic thaw ponds at all sites were thermally stratified, consistent with previous observations in summer (Laurion et al., 2010). There were large vertical gradients in chemical properties, with a surface oxic layer (epilimnion) overlying low oxygen bottom waters (hypolimnion). These gradients provided a wide range of potential bacterial habitats within a single pond. The ponds contained diverse bacterial assemblages, with the number of OTUs (99–307 per sample) overlapping that reported for much deeper, stratified water bodies. For example, 280–425 OTUs were reported from a meromictic lake in the High Arctic using the same primers for tag pyrosequencing the 16S rRNA gene (Comeau et al., 2012), and 67–223 OTUs were reported for thermally stratified German lakes (Garcia et al., 2013). The Shannon and Simpson diversity indices in the thaw ponds were greater than those reported from German lakes, which were all less than 4.1 for the Shannon index and 0.36 for the Simpson index (Garcia et al., 2013). However, the taxonomic richness of these thaw pond communities was much lower than that in soils. For example, 4781–6231 OTUs were reported in 10 g samples of German grassland soils (Will et al., 2010) and 1496–1857 OTUs in the same quantity of High Arctic soil crusts (Steven et al., 2013; singletons excluded). Previous studies have documented this large disparity between lakes and soils (Lozupone and Knight, 2007; Tamames et al., 2010), suggesting fundamental difference in microhabitats in the highly structured matrix of soils relative to aqueous planktonic environments. Given the large potential input of soil particles and their associated bacteria from permafrost degradation, higher levels of diversity and species richness might have been expected in these waters. However, the total number of OTUs (1296) for our entire study including all 22 samples was 17% of that observed in the High Arctic soil crust study (7432), and within the range for other aquatic ecosystems.

The Shannon and Simpson diversity indices showed that all samples were equally diverse, with no significant differences among valleys, depths, or size fractions. Steven et al. (2013) similarly observed in their soil crust analyses of six sites in the High Arctic that differences in diversity indices among sites were small relative to comparisons with other systems, including Antarctic soils. It would be of great interest to compare bacterial diversity in these northern thaw ponds with the shallow ponds of Antarctica, but to our knowledge such data are lacking. The significant difference of Chao1 index between KWK and BGR valleys indicated that species richness was higher at the lower latitude site. Species richness values for the surface waters were the same order of magnitude for the three valleys, however, there was a significant interaction of species richness between sites and depth, with species richness of bottom water increasing from BGR to KWK and SAS. This also corresponds to a gradient in DOC, with highest concentrations in the SAS ponds, and may reflect differences in bacterialproductivity.

RNA versus DNA templates have been used to distinguish between active and inactive cells (Jones and Lennon, 2010) and to assess potential growth rates (Campbell and Kirchman, 2013). 16S rRNA is thought to provide an estimate of ‘potentially active bacteria’ (Blazewicz et al., 2013), which would be a subset of the community represented in the environmental DNA. The latter may include dead cells, spores and even free DNA that remains intact in the dark, cold, polar environment (e.g., Charvet et al., 2012). For this reason it is thought that the use of DNA can lead to overestimation of diversity, while rRNA may provide a more conservative and accurate estimation of diversity.

### Bacterial Dominants

For the overall data set, the dominant phylum was Proteobacteria and β-proteobacteria the dominant Class, as in many freshwater ecosystems (Newton et al., 2011). Two genera within that Class were particularly abundant, each on average representing around 10% of the total number of reads: *Polynucleobacter* and *Variovorax.* Both have attributes that make them well-suited to the heterogeneous combination of allochthonous (terrestrial) and autochthonous (aquatic) organic carbon compounds that are likely to occur in these waters. *Polynucleobacter* is a cosmopolitan genus that often dominates planktonic freshwater communities. It produces extremely small cells that are capable of growing aerobically on a wide range of complex media (Hahn, 2003), although genomic analyses of free-living and symbiotic strains of *Polynucleobacter necessarius* indicate a small genome size and the absence of certain functions, including an inability to use sugars as a carbon and energy source (Boscaro et al., 2013).

*Variovorax* includes taxa with diverse nutritional and energy acquisition strategies, including the breakdown and use of a wide variety of plant-derived molecules, as well as denitrification, sulfate reduction, and autotrophic CO_2_ fixation. Genomic analysis of a plant-associated *Variovorax paradoxus* strain has shown that it has a remarkable combination of features for both heterotrophic and autotrophic lifestyles, and although it lacks the genes for methane monooxygenase, it is known to enhance the activity of methanotrophs in consortia (Han et al., 2011; and references therein). This strain also has three genes encoding aerobic carbon monoxide dehydrogenase, which could be of value given that high levels of carbon monoxide are known to be produced by photochemical reactions in high latitude, DOC-rich waters (Xie et al., 2009).

Subarctic thaw ponds emit methane (Laurion et al., 2010), and the presence and diversity of methanotrophic bacteria is of particular interest. Consist with our hypothesis, methanotrophs were found in all samples, and included three of the most abundant OTUs: two strains of *Methylobacter*, and one OTU with affinities to the family Methyloccocaceae. The methanotrophic bacteria all belonged to Type I methanotrophs which are in the γ-proteobacteria and the Verrucomicrobia. No bacteria belonging to Type II methanotrophs in the α-proteobacteria were identified. Methanotrophic communities are known to be sensitive to temperature, with Type I methanotrophs developing under low temperature conditions (Wartiainen et al., 2003; Börjesson et al., 2004; Wagner et al., 2005; Graef et al., 2011) and Type II under higher temperatures (Mohanty et al., 2007). The absence of Type II methanotrophs from even the warmer surface waters of the thaw ponds in summer implies selection by the low temperature conditions that prevail throughout the water column during most of the year.

Methanotrophs contributed a relatively high percentage of the total number of sequences in the thaw ponds, with a median of 4.9%, and a maximum of 27% in the bottom waters of KWK23. This is particularly high relative to other lakes, for example up to 2% in a meromictic lake in the High Arctic (Comeau et al., 2012) and only up to 3% of sequences in the plankton of eutrophic Lake Pluβsee in Germany (Eller et al., 2005). This high relative abundance is closer to that observed in tundra soils (Vecherskaya et al., 1993) and anoxic lake sediments (Costello and Lindström, 1999), indicating the biogeochemically distinct nature of thaw pond ecosystems, with continuously high inputs of methane as a bacterial energy source.

Two groups of bacterial phototrophs were conspicuous members of the thaw pond assemblages: cyanobacteria and Chlorobi. The presence and abundance of sequences for these taxa varied among the samples, in part associated with depth and valley specific characteristics (see below). Cyanobacteria are often dominants of high latitude aquatic ecosystems, especially mat-forming Oscillatoriales and picoplanktonic taxa in the genus *Synechococcus* (Vincent, 2000). In the thaw pond data set, Oscillatoriales were little represented (with the exception of the bottom of SAS1B), as might be expected in a planktonic environment, which was in contrast to High Arctic soil crusts where Oscillatoriales are among the dominants. Mat-forming taxa are unlikely to thrive on the sediments of the subarctic thaw ponds given the high turbidity and the poor penetration of photosynthetically active radiation to the bottom (Watanabe et al., 2011). The picocyanobacterial group Synechococcales was represented in many of the ponds, although accounted for only 0.7% of the total bacterial reads. A surprisingly more abundant cyanobacterial group in two of the ponds was the Nostocaceae, with strong affinities (>99%) to the nitrogen-fixing taxon *Dolichospermum curvum* (formerly known as *Anabaena curvum*). Colonial cyanobacteria are largely absent from the plankton in other waters of the north and south polar regions (Vincent, 2000), again underscoring the distinctive properties of permafrost thaw ponds as an ecosystem type.

*Pelodictyon* was the fourth most abundant OTU in the overall data set, accounting for 3.3% of all sequences. This green sulfur bacterium (Chlorobi) is often observed under anoxic conditions in stratified freshwater lakes at depths where there is sufficient light for photosynthesis as well as high concentrations of its electron donor hydrogen sulfide. Chlorobi were earlier reported from 16S rRNA gene clone libraries of the KWK valley (Rossi et al., 2013), and their importance in the deep low- oxygen waters in many of the lakes suggest that anaerobic photosynthesis likely contributes significantly to the production of these subarctic ecosystems.

### Attached and Free-Living Bacteria

In a wide range of marine systems, particle-attached, and free-living bacterial communities are morphologically and phylogenetically distinct (e.g., Crump et al., 1999; Lapoussière et al., 2011; Mohit et al., 2014). Similarly in some freshwaters, for example Lake Erie (Mou et al., 2013) and Lake Bourget (Parveen et al., 2011) differences in attached and free-living bacterial communities have been reported. Here, we found little difference between the communities from our large and small fractions, which should have selected for attached and free-living bacterial communities respectively. The lack of difference in some of the samples may have been due to extremely small particulates in some of these ponds (ca. 1 μm; Watanabe et al., 2011) that would have passed through the 3-μm filter along with their attached bacterial flora, masking any differences. However, Chao1 diversity was significantly greater in the >3 μm size class and this would be more consistent with the >3-μm filters retaining both attached and many free-living bacteria if the filters became blocked. Our inability to filter more than 500 mL of sample suggest this latter explanation is more likely, and it is also consistent with the presence of picocyanobacteria (Synechococcales) in some of the large fraction samples, notably from BGR1 (**Figure 5**). Irrespective of the cause, more detailed size fractionation or a microscopy approach would be needed to accurately resolve the difference between particle-attached and free-living bacteria in these highly turbid ponds.

### Depth Gradients and Bacterial Community Composition

The thaw ponds were stratified with pronounced gradients in temperature and oxygen. Although the ponds are shallow, the high concentrations of CDOM and small wind fetch mean that they stratify early in spring and remain so over the summer (Watanabe et al., 2011). In fact, there is evidence that some ponds may not mix to the bottom at all in some years (Laurion et al., 2010). Within valleys, surface and bottom communities clustered well-apart from each other, with the exception of the SAS site where surface waters were depleted in oxygen compared to the other sites (**Figure 5** and **Table 1**). In terms of community composition, Actinobacteria were often poorly represented at the bottom of all ponds, consistent with their preference for more oxygenated waters (Allgaier and Grossart, 2006; Taipale et al., 2009). Anaerobic sulfate reducers, including the δ-proteobacteria *Geobacter, Anaeromyxobacter* and *Desulfovibri,* were found in small proportions in the bottom of ponds. Other bacterial dominants with matches to the family Chitinophagaceae (Bacteroidetes) including *Sediminibacterium* have the ability to produce H_2_S (Qu and Yuan, 2008), consistent with an active sulfur cycle and anaerobic conditions.

Some of the largest depth-dependent differences were for the phototrophic taxa, in keeping with the rapid attenuation of photosynthetically available radiation in these waters (Watanabe et al., 2011), as well as the depth variations in chemical properties of the ponds. The anoxygenic phototroph *Pelodictyon* was one of the most abundant OTUs, but was restricted to low oxygen bottom waters, as expected, and was absent from the more oxygenated BGR sites. This photosynthetic sulfur bacterium is adapted to low light and anoxic, H_2_S-containing waters (Gich et al., 2001). Cyanobacteria in contrast, tended to be in the upper euphotic zone, with the KWK sites especially having a greater relative representation of cyanobacteria in the surface compared to the bottom of the ponds. The most striking difference was in the surface waters of KWK6 where Nostocaceae were the dominant cyanobacterial group, indicating the likely growth of colonial, potentially nitrogen-fixing species in these surface waters. However, for other ponds the large fraction of BGR1 bottom sample was dominated by Synechococcales, possibly indicating the growth of smaller celled cyanobacteria able to maintain populations in the better illuminated bottom waters of BGR1, where concentrations of light-attenuating materials (Chl-*a*, DOC, TSS) were less than most of the other ponds. Oscillatoriales, which are filamentous, were found in the bottom of another pond, SAS1B, possibly indicating the sedimentation of aggregates of filaments or sections of mat from shallower depths.

In the KWK and SAS waters, methanotrophs were more abundant in the bottom waters, consistent with the earlier reported profiles of methane in the ponds. For example in KWK23, where the difference between surface and bottom samples was particularly striking, methane concentrations increase sharply at the bottom of the pond by several orders of magnitude (Laurion et al., 2010). Methanotrophs are aerobic and unable to sustain growth under completely anoxic conditions (Chowdhury and Dick, 2013), and our observations imply that the deep pond habitat provides a favorable combination of high methane and adequate oxygen. Interestingly, in the BGR ponds where there was less difference between surface and bottom oxygen, there was a higher proportional abundance of methanotrophs in the surface waters; this might suggest dependence on methane production from decomposition of macrophytes in the littoral zone, or via plant–microbe interactions in this region (Laanbroek, 2010).

### Spatial Variation and Landscape Gradients

Our results showed a clustering of ponds according to the individual valleys (**Figure 5**), implying environmental filtering of community composition based on landscape related properties (**Figure 6**). The dbRDA pointed to DOC concentrations and pH as controlling factors, both of which are known to influence bacterial community structure (e.g., Lindström et al., 2005; Fierer and Jackson, 2006). The higher pH in the BGR ponds separated them from the ponds in the other two valleys, although not all microbial groups followed expected relationships. Verrucomicrobia have been associated with low pH conditions (Lindström et al., 2005), but this group was relatively abundant in the BGR pond with the highest pH, and poorly represented in the SAS pond with the lowest pH. However, the pH range in this study was narrower than in Lindström et al. (2005).

Dissolved organic matter origin and source has been reported to influence bacterial community structure and function (Kritzberg et al., 2006). DOC had the greatest influence on community composition at the SAS site, where the ponds originated from organic palsas and DOC concentrations were higher and likely different in composition from the DOC at the other two valleys, where the ponds were formed by lithalsa thawing. The permafrost thaw gradient was also associated with the availability of DOC. The BGR valley is surrounded by discontinuous permafrost and >50% of the soil carbon would be frozen, and not available for degradation. In contrast, the southern, more degraded KWK and SAS valleys, ponds would be influenced by the eroding permafrost (Bouchard et al., 2014), and the input of allochthonous DOC would be more substantial. Photochemical breakdown of some of the more recalcitrant soil organic materials to lower molecular weight compounds (Laurion and Mladenov, 2013) may additionally enhance substrate availability in these waters.

## Conclusion

Permafrost thaw lakes and ponds are a prominent feature of the northern landscape and are strong emitters of greenhouse gasses. Because of their abundance on the landscape and wide distribution they are also useful for investigating the influence of large scale versus small scale environmental gradients. We found that permafrost gradients influenced the landscape properties, in turn driving bacterial communities composition. Within a pond, the physico-chemical stratification creates oxygen gradients that favor different microbes. In permafrost thaw lakes, the variety of allochthonous substrates derived from terrestrial vegetation and soils, and autochthonous sources including oxygenic photosynthesis by cyanobacteria, microalgae and macrophytes and anoxygenic photosynthesis by green sulfur bacteria, likely provide a heterogeneous range of organic substrates available to diverse heterotrophic taxa. Methanotrophs were among the most abundant sequences at most sites, indicating the potential importance of methane as a bacterial energy source in these waters. Their activities likely reduce the net emission of methane, in the process contributing to the CO_2_ eﬄux from these ecosystems. The functionally diverse bacterial taxa in these abundant ‘biogeochemical hot spots’ across the subarctic landscape likely have a strong effect on the net emission of both greenhouse gasses, as the result of their metabolism of organic carbon from multiple sources. Ancient permafrost soils are now being increasingly thawed, eroded and mobilized as a result of the rapid warming of the North. The diverse bacterial communities identified here will likely assure that at least part of these new transfers from land to water are ultimately converted to CO_2_ and released to theatmosphere.

## Author Contributions

This study was conceived and designed by SC, WV and CL, field work was conducted by SC, WV and JC, laboratory analysis was by SC, bioinformatics analysis was by SC, JC and CL, and all authors contributed to the writing of themanuscript.

## Conflict of Interest Statement

The authors declare that the research was conducted in the absence of any commercial or financial relationships that could be construed as a potential conflict of interest.
