# Supplementary material for: Bacterial community structure across environmental gradients in permafrost thaw ponds: methanotroph-rich ecosystems
Source: Front Microbiol. 2015 Mar 18;6:192. doi: 10.3389/fmicb.2015.00192 (PMC4396522; doi:10.3389/fmicb.2015.00192)
Supplement: Supplementary file 1 [file Table_1.DOCX]

**Bacterial community structure across environmental gradients in permafrost thaw ponds and implications for greenhouse gas emissions**

Sophie Crevecoeur^1, 2, 3*^, Warwick F. Vincent^1,2^, Jérôme Comte^1, 2, 3^ and Connie Lovejoy^1,3,4^

^1^Département de biologie and Takuvik Joint International Laboratory, Université Laval, Québec, QC, Canada
^2^Centre for Northern Studies (CEN), Université Laval, Québec, QC, Canada
^3^Institut de Biologie Intégrative et des Systèmes (IBIS), Université Laval, Québec, QC, Canada
^4^Québec Océan, Université Laval, Québec, QC, Canada

***Correspondence: Sophie Crevecoeur, Université Laval, Département de biologie, Pavillon Alexandre Vachon, 1045 Avenue de la Médecine,** QC, G1V 0A6, Canada, [sophie.crevecoeur.1@ulaval.ca](mailto:sophie.crevecoeur.1@ulaval.ca)

Supplemental online material

Supplemental Table 1. Sequencing results of the number of reads and number of OTUs and diversity indexes calculated with QIIME for each sample. Samples are designated by pond name followed by the position in the water column (surface, S; bottom, B), and then by fraction (small, S; large, L).

| Sample | Reads | OTUs | Shannon | Simpson | Chao1 |
| --- | --- | --- | --- | --- | --- |
| SAS1B-S-S | 3837 | 204 | 5.17 | 0.93 | 218 |
| SAS1B-B-S | 4384 | 198 | 4.41 | 0.89 | 223 |
| SAS2A-S-S | 3242 | 169 | 4.07 | 0.87 | 195 |
| SAS2A-B-S | 4063 | 163 | 3.91 | 0.84 | 188 |
| KWK1-S-S | 3071 | 116 | 5.02 | 0.94 | 128 |
| KWK1-B-S | 10562 | 307 | 4.03 | 0.72 | 340 |
| KWK1-S-L | 6114 | 185 | 5.44 | 0.95 | 199 |
| KWK1-B-L | 7807 | 291 | 4.80 | 0.82 | 315 |
| KWK6-S-S | 3832 | 182 | 5.82 | 0.96 | 213 |
| KWK6-B-S | 3639 | 182 | 5.16 | 0.94 | 222 |
| KWK6-S-L | 5856 | 202 | 4.98 | 0.91 | 223 |
| KWK6-B-L | 4916 | 238 | 5.30 | 0.94 | 256 |
| KWK23-S-S | 5502 | 186 | 5.60 | 0.96 | 219 |
| KWK23-B-S | 3577 | 223 | 5.18 | 0.91 | 241 |
| BGR1-S-S | 4374 | 127 | 4.82 | 0.93 | 150 |
| BGR1-B-S | 3922 | 102 | 4.48 | 0.89 | 114 |
| BGR1-S-L | 6058 | 261 | 6.09 | 0.97 | 275 |
| BGR1-B-L | 5993 | 209 | 5.43 | 0.94 | 222 |
| BGR2-S-S | 3922 | 99 | 4.13 | 0.89 | 110 |
| BGR2-B-S | 4164 | 106 | 3.86 | 0.85 | 116 |
| BGR2-S-L | 6936 | 204 | 4.26 | 0.80 | 235 |
| BGR2-B-L | 6708 | 141 | 4.02 | 0.85 | 153 |
